# Nickel contamination after minimally-invasive repair of pectus excavatum persists after bar removal

**DOI:** 10.1371/journal.pone.0275567

**Published:** 2022-10-10

**Authors:** Caroline Fortmann, Thomas Goeen, Norman Zinne, Soeren Wiesner, Benno M. Ure, Claus Petersen, Joachim F. Kuebler

**Affiliations:** 1 Department of Pediatric Surgery, Hannover Medical School, Hannover, Germany; 2 Institute and Outpatient Clinic of Occupational, Social and Environmental Medicine, Friedrich-Alexander-Universität Erlangen-Nürnberg, Erlangen, Germany; 3 Department of Cardiothoracic, Transplantation and Vascular Surgery, Hannover Medical School, Hannover, Germany; 4 Institute for Biometry, Hannover Medical School, Hannover, Germany; Sapienza University of Rome: Universita degli Studi di Roma La Sapienza, ITALY

## Abstract

**Background:**

Minimally-invasive repair of pectus excavatum (MIRPE) has been shown to be associated with high release of trace metals into patient’s body. The aim of our study was to analyze the kinetics of metal contamination after MIRPE and after bar removal.

**Methods:**

We prospectively assessed nickel and chromium changes in blood, urine, and local tissue in patients undergoing MIRPE with stainless-steel bar(s). Baseline samples were taken prior to surgery, further samples were taken at six defined time points until 30 months after bar removal. Clinical symptoms were evaluated at the time of every sample collection.

**Results:**

28 patients were included (mean age 16.4 years). At four weeks after MIRPE and persisting up to bar removal, we found significantly elevated trace metal levels in blood and urine. Tissue nickel and chromium levels were significantly elevated at the time of bar removal. After bar removal, the concentration of trace metal in urine and the concentration of chromium in plasma decreased gradually. In contrast, nickel levels in blood further increased. Five patients showed irritative symptoms after MIRPE, all symptomatic patients had elevated metal levels.

**Conclusions:**

Following MIRPE, we found a rapid systemic increase of nickel and chromium. Our data indicate that trace metal release could cause irritative symptoms. The prolonged elevated systemic nickel levels beyond bar removal necessitate further investigations of the long-term side effects of MIRPE.

## Introduction

Minimally-invasive repair of pectus excavatum (MIRPE) is the goldstandard treatment of “funnel chest” [1]. Under thoracoscopic guidance, one or more metal bars are placed substernally to elevate the sternum and are fixed on the lateral chestwall if necessary. After three years, when the chestwall is permanently remodeled bars should be removed.

In recent years, a high incidence of allergic symptoms after MIRPE has been reported [2–4]. We observed extremely elevated nickel and chromium levels in local tissue at bar removal. Additionally, we identified significantly elevated nickel levels in blood and chromium levels in urine of patients undergoing bar removal [5]. All patients who had irritative symptoms during the first weeks after MIRPE had elevated trace metal levels at the time of bar removal. Other authors have found significantly elevated chromium levels in blood of patients after a mean time of 13 months after MIRPE [6] and at bar removal [7].

In orthopedic surgery, numerous studies on metal release after implantation of joint replacement prostheses with a metal-on-metal surface have been published [8, 9]. Articulating surfaces are known to produce wear particles, and all implanted metallic devices corrode when in contact with body fluids and tissue [10]. Hip arthroplasty has led to a significant local and systemic contamination with chromium and cobalt ions, resulting in aseptic loosening and early implant failure [11]. It is still unclear whether metal allergy leads to failing prostheses or if implant failure results in a metal allergy [12, 13].

The kinetic of the trace metal contamination after pectus bar implantation is still unknown, as is its relation with symptoms. Moreover, it is unknown whether the contamination stops after bar removal.

Therefore, we analyzed the kinetics of nickel and chromium levels after MIRPE and after bar removal.

## Materials and methods

The institutional review board approved this study (No. 6659), and all patients and legal guardians gave their informed consent. We consecutively included all patients undergoing MIRPE at the department of pediatric and thoracic surgery of the Hannover Medical School from March 2015 until February 2017.

### Patients’ characteristics, operative technique, and follow-up

Patients’ characteristics (age, sex, number and size of bars) were collected prospectively. Prior to MIRPE, patients were asked about metal allergy. A stainless-steel allergy test plate was applied in patients with a positive history of metal allergy. Patients with a positive test result were excluded from the study. MIRPE was performed as previously described [5]. Briefly, one or more stainless steel bars were placed underneath the sternum under thoracoscopic guidance, and stainless-steel stabilizers were affixed on both ends of the bar. Slight movements between stabilizers and bar were possible, and a pin at the far end of the bar prevented stabilizer dislocation. Intraoperatively, a pectus bar tabletop bender was used for individual shaping of the bar. Following MIRPE, 1.8 g clindamycine was given for ten days as hospital’s standard even without clinical evidence in the literature, started intravenously and switched to oral administration after three to four days. Follow-up was performed after four weeks as well as one and two years postop. Patients were interviewed by phone by one doctor of the study team, and clinical symptoms were evaluated. In case of symptoms, patients were seen in the outpatient clinic. Bar removal was recommended three years after MIRPE. Further follow-up was carried out one year and up to three years following bar removal.

### Definition and treatment of symptoms

As irritative symptoms, we differentiated rash, pleural effusion, seroma, local swelling, wound healing disorder, persistent pain, and lassitude. Rash was defined as diffuse redness at the level of the bar. Pleural effusion was diagnosed on x-ray and sonography. Seroma and swelling were examined using ultrasound. Definition of wound healing disorder included local wound dehiscence with negative bacterial cultures. Persistent pain was defined as pain lasting six weeks after MIRPE requiring analgesic therapy. When patients reported impossibility of completing daily activities, fatigue and lassitude were diagnosed. This symptom was considered as irritative symptom when it occurred in combination with a local reaction. Our treatment concept of irritative symptoms included 50mg prednisolone for one to two weeks, which was thereafter tapered over several weeks. Additionally, we prescribed 1.8g clindamycine daily for one to two weeks.

### Bar composition

According to the producer (MedXpert, Germany), the bar consists of 1.4441 ASTM F 138 stainless steel. The main components aside from iron are chromium (18%) and nickel (15%). Additional elements are molybdenum (3%), manganese (2%) and other metals and semimetals (silicium, cobalt, copper and aluminum, each <1%).

### Method of sample collection for trace metal analysis

We focused on nickel and chromium due to their high content in the bar, their allergic potential, and the metal contamination reported after hip arthroplasty [8, 9]. We did not analyze cobalt levels, as we have previously shown no significant increase in such levels after MIRPE [5]. Utilized collection tubes and cannulas were free of any trace metal contamination. Local subcutaneous tissue was collected intraoperatively before implantation. During bar explantation, local tissue was extracted directly next to the stabilizer. Blood and urine samples were taken before surgery and at the follow-up time points.

### Trace metal analysis

Analysis of chromium and nickel was performed at the Institute of Occupational, Social and Environmental Medicine of the Friedrich-Alexander-Universität Erlangen-Nürnberg, as previously described [5]. Briefly, the chromium and nickel content present in patients’ tissue, urine, and blood was assessed using an inductively coupled plasma mass spectrometer (ICP-MS) with collision cell (Agilent 7500cx). Monitoring of the ion mass 52 (chromium) and 60 (nickel) was used for quantification.

### Appraisal of trace metal levels

Reference values for trace metal levels in blood and urine have been published by the *DFG Commission for the Investigation of Health Hazards of Chemical Compounds in the Work Area* and indicate the upper metal level in the general German population (nickel in blood 0.5μg/l, nickel in urine 3μg/l, chromium in plasma 0.4μg/l, chromium in urine 0.6μg/l) [14]. Trace metal contamination was considered when the individual levels were above the reference values. Intoxication was defined as contamination in combination with irritative symptoms.

### Data analysis

Statistical analysis was performed using SigmaStat^®^ by Jandel Scientific. Data were reported as mean ± standard error of the mean and range for continuous variables and as percentages for categorical variables. Mean tissue levels at the time of bar removal were compared to mean levels prior to MIRPE using the paired t-test. The postoperative intraindividual changes in blood and urine from preop trace metal levels were calculated. Statistical analyses were performed using the one-way analysis of variance on ranks and Dunn’s method for posthoc analysis of each follow-up time point to baseline. A p-value of less than 0.05 was considered to be statistically significant.

## Results

37 patients underwent MIRPE between March 2015 and February 2017 and were enrolled. In these patients, bar removal was performed from March 2018 until March 2020. Eight patients had to be excluded from metal analysis due to incomplete data at bar explantation. In one patient, permanent and drug-resistant irritative symptoms led to early bar removal after five months; therefore this patient was also excluded and analyzed separately. The remaining 28 patients were included for statistical analysis.

### Patients’ characteristics and operative specifics

Mean age at the time of MIRPE was 16.4 years (± 0.7 years; range 13 to 28 years) and 92.9% were male. No patient reported a history of metal allergy prior to MIRPE. We used one bar in 17 patients (60.7%), two bars in ten patients (35.7%) and three bars in one patient (3.6%). Mean length of the bars was 11 inches (± 0.3 inch; range 9 to 15 inches). Bar removal was performed after a mean time of 36 months (± 0.75 months; range 30 to 52 months). The subsequent blood and urine collection was scheduled after a mean time of one year (11 months ± 0.6 months; range 8 to 17 months) following bar removal. Final trace metal levels were sampled at a mean time of 30 months (± 2.1 months; range 20 to 38 months) following explantation.

### Trace metal values

In all local and systemic samples, we found a significant increase in mean nickel and chromium levels at bar removal compared to values before MIRPE (Table 1).

**Table 1:** Mean trace metal levels over the study period.

	preop	4 weeks postop	1 year postop	2 years postop	bar removal	1 year after bar removal	30 months after bar removal
**Nickel in blood** (μg/l)	*0*.*23*	*0*.*49*	0.51	0.70	1.04	1.12	1.75
**Nickel in urine** (μg/l)	*1*.*80*	*2*.*64*	6.00	5.25	3.81	*1*.*09*	*1*.*01*
**Chromium in plasma** (μg/l)	*0*.*20*	*0*.*12*	0.70	1.46	1.01	0.77	0.57
**Chromium in urine** (μg/l)	*0*.*27*	*0*.*43*	1.79	2.45	2.95	1.15	*0*.*54*
**Nickel in tissue** (μg/g)	1.9				303.0		
**Chromium in tissue** (μg/g)	3.5				916.46		

Values below the reference value are written in italics.

#### Nickel levels

**Fig 1:**
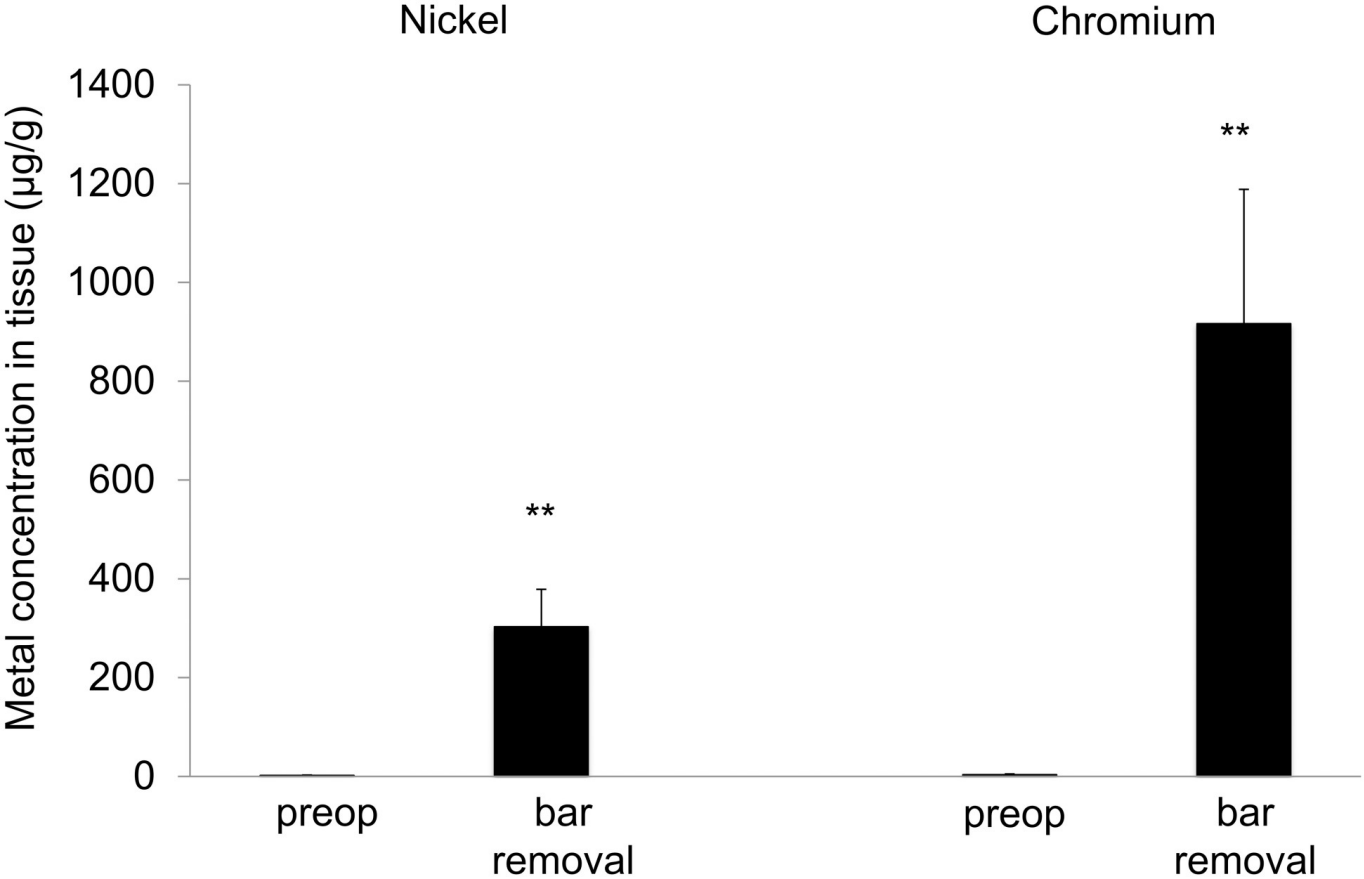
Nickel and chromium levels in local tissue before MIRPE and at time of bar removal. Mean concentrations of nickel and chromium in tissue, error bars show standard error of the mean. The differences between concentrations preoperatively and at bar removal were statistically significant (** p<0.*01*).

**Fig 2:**
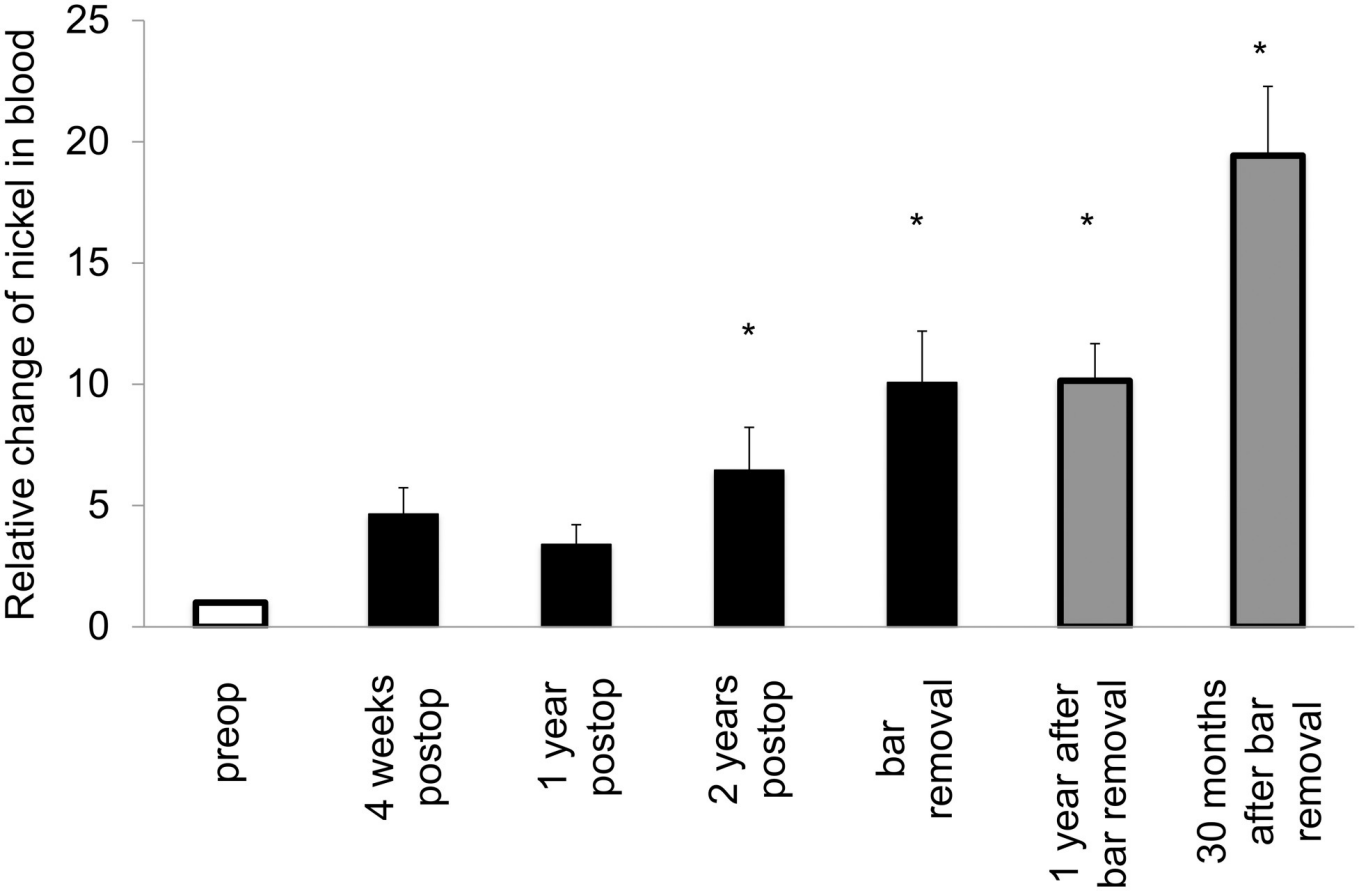
Intraindividual changes of systemic trace metal levels following MIRPE. Mean intraindividual changes of systemic trace metal concentrations, error bars show standard error of the mean. The difference overall was statistically significant (*p<0*.*001*) as well as the differences between the particular groups and preop (*p<0.*05*).

**Fig 3:**
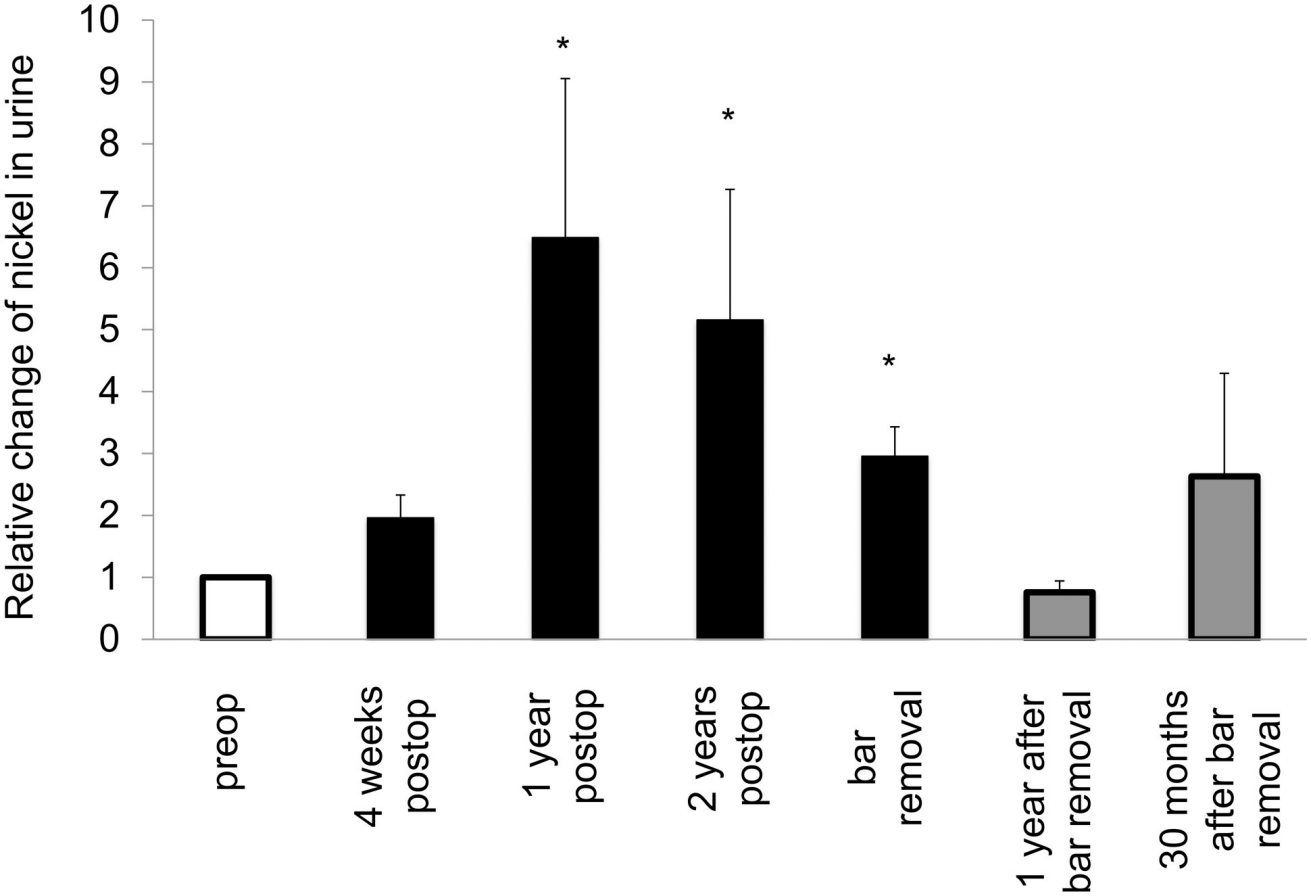
Intraindividual changes of systemic trace metal levels following MIRPE. Mean intraindividual changes of systemic trace metal concentrations, error bars show standard error of the mean. The difference overall was statistically significant (*p<0*.*001*) as well as the differences between the particular groups and preop (*p<0.*05*).

Mean nickel levels in blood were increased in the whole postoperative period and persistently raised after bar removal (Table 1). Mean values exceeded the reference value one year postoperatively and thereafter. Mean nickel levels in urine also increased until bar removal and were higher than the reference value after one year. Following bar removal, nickel levels in urine decreased and were below the reference value (Table 1). Tissue nickel levels were significantly increased at bar removal (Table 1 and Fig 1). Systemic intraindividual changes compared to baseline values confirmed a significant increase postoperatively (Figs 2 and 3). The intraindividual changes of nickel levels in urine 30 months after bar removal appear to rise but this is explained by slight variations in the patient group who kept the follow-up appointment. Due to very low nickel levels in urine before MIRPE, follow-up levels appear to increase but are still underneath the reference value.

#### Chromium levels

**Fig 4:**
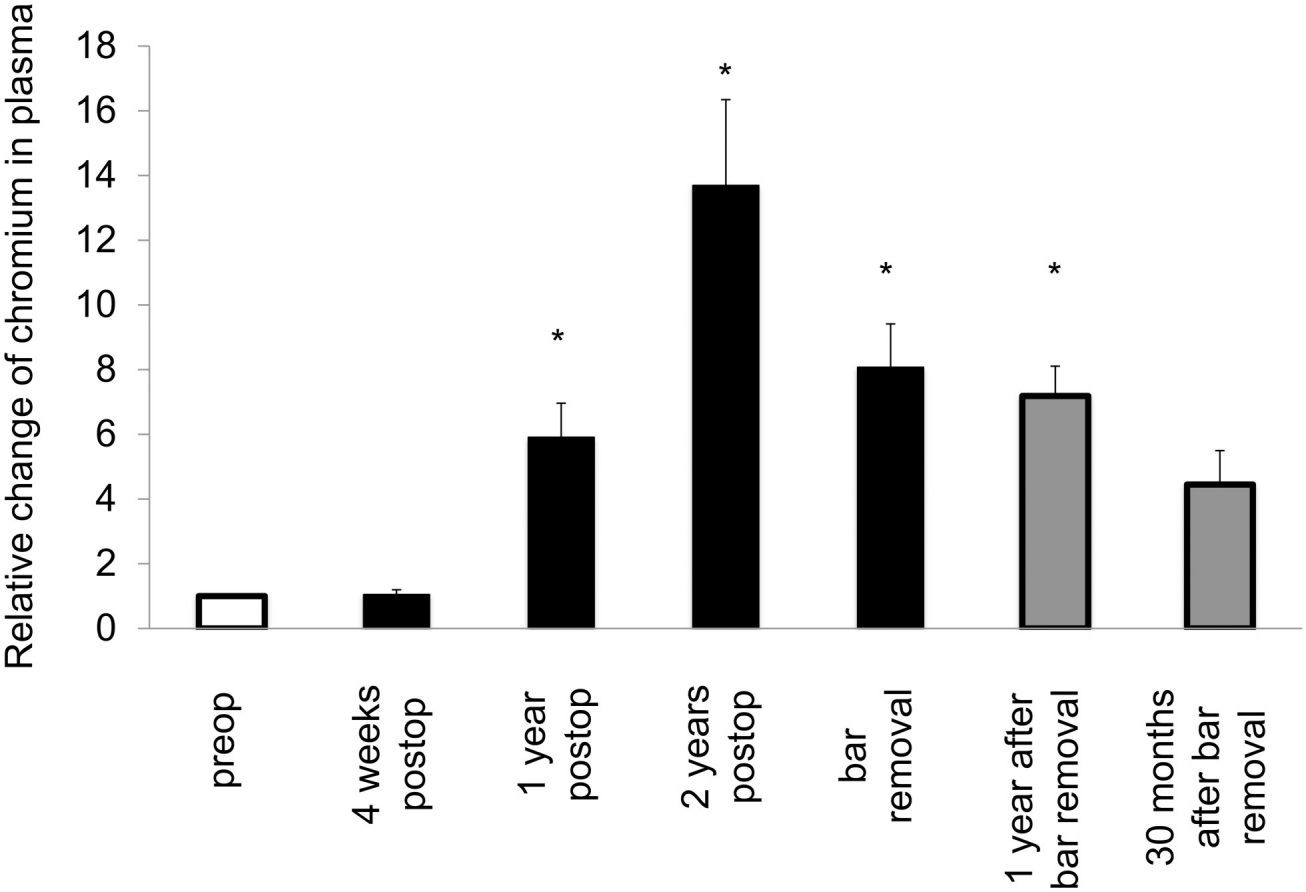
Intraindividual changes of systemic trace metal levels following MIRPE. Mean intraindividual changes of systemic trace metal concentrations, error bars show standard error of the mean. The difference overall was statistically significant (*p<0*.*001*) as well as the differences between the particular groups and preop (*p<0.*05*).

**Fig 5:**
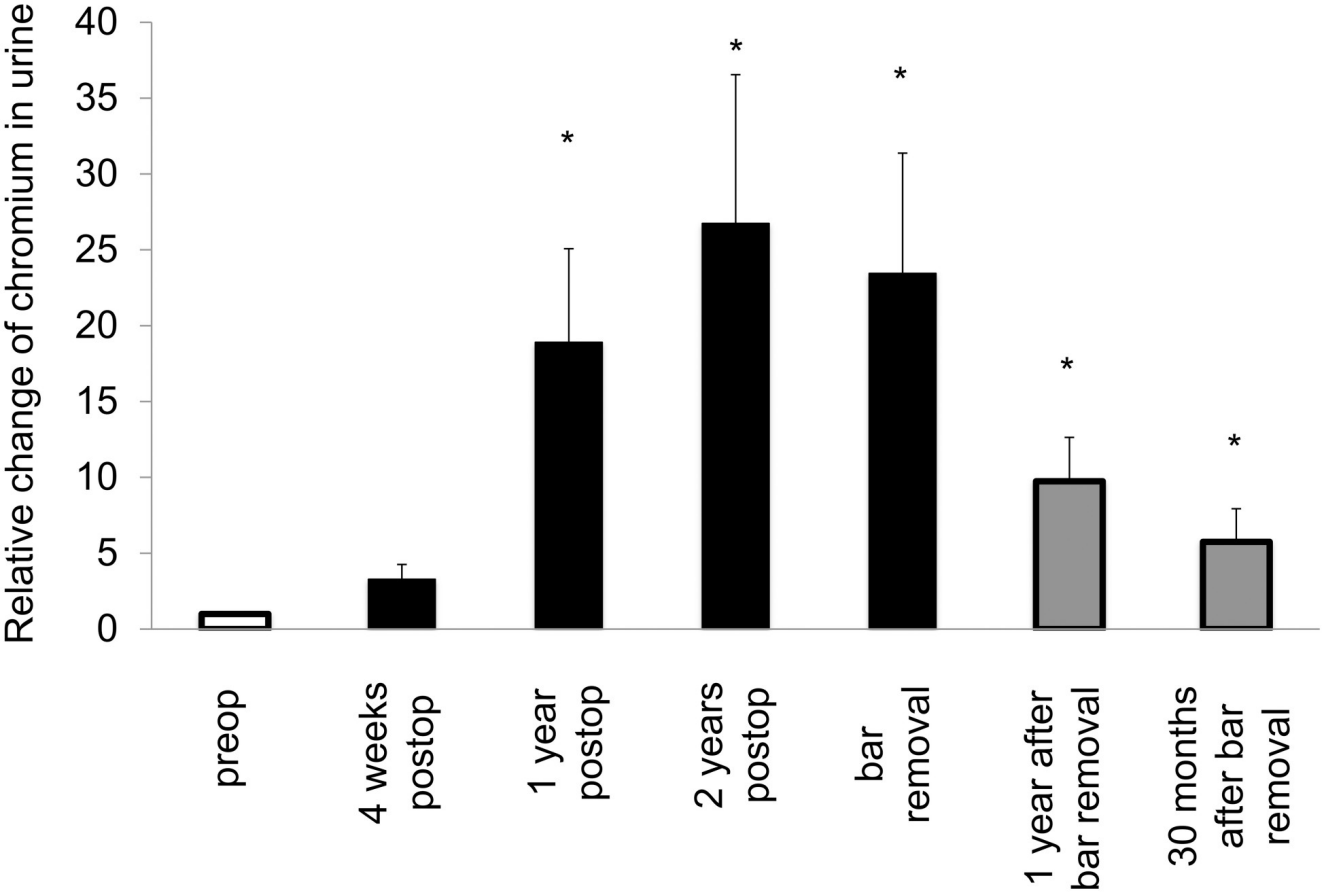
Intraindividual changes of systemic trace metal levels following MIRPE. Mean intraindividual changes of systemic trace metal concentrations, error bars show standard error of the mean. The difference overall was statistically significant (*p<0*.*001*) as well as the differences between the particular groups and preop (*p<0.*05*).

Mean chromium levels in plasma showed an increase after one year, with values above the reference value. Bar removal caused a decrease; however, values were still above the reference value (Table 1). Mean chromium levels in urine steadily rose until bar removal; the reference value was exceeded one year post MIRPE and thereafter. Following bar removal, mean values decreased and lay below the reference value 30 months later (Table 1). Tissue chromium values at bar removal showed a significant increase in comparison to preop (Table 1 and Fig 1). The systemic intraindividual changes postoperatively were significantly different than the baseline (Figs 4 and 5).

Comparison of systemic trace metal levels between patients with one bar and patients with more than one bar showed no significant differences. However, tissue levels at bar removal were significantly lower in patients with multiple bars (*p<0*.*01*). This finding could be explained by a greater area on which forces from the reshaped chestwall are distributed. This could result in lower stress on each bar and stabilizer.

### Follow-up

Due to problems with patients’ compliance, follow-up data were incomplete (82% after four weeks, 82% after one year, 57% after two years, and 61% following bar removal).

### Appearance and treatment of symptoms

Of the total 37 patients enrolled, five (13.5%, all male) developed irritative symptoms during the first weeks after bar implantation (mean time after MIRPE: 21 days; ± 7.3 days, range 10 days–five weeks). Only one patient needed to be excluded from statistical analysis due to bar removal ahead of schedule. Symptoms in the remaining four patients consisted of local rash (three patients), lassitude (two patients), pain (two patients), and local swelling (one patient). Treatment included 50mg prednisolone and 1.8g clindamycine daily in two patients, while one patient only received clindamycine. The symptoms disappeared after a few days. One patient refused controls between surgeries and presented for bar removal with a typical rash without any treatment over the years. His rash vanished rapidly after bar removal.

No irritative reactions occurred during the further follow-up. In all symptomatic patients, we found elevated trace metal levels post MIRPE (nickel in blood in three patients, nickel in urine in two patients, chromium in plasma in two patients, chromium in urine in three patients). However, in all other patients, we measured elevated trace metal values without the appearance of irritative symptoms. There was no difference of trace metal levels between symptomatic and asymptomatic patients.

At the time of bar removal, we observed a subcutaneous discoloration in most patients on top of the bar where the stabilizers were placed, which could be metal debris.

### Therapy-resistant irritative symptoms

**Fig 6:**
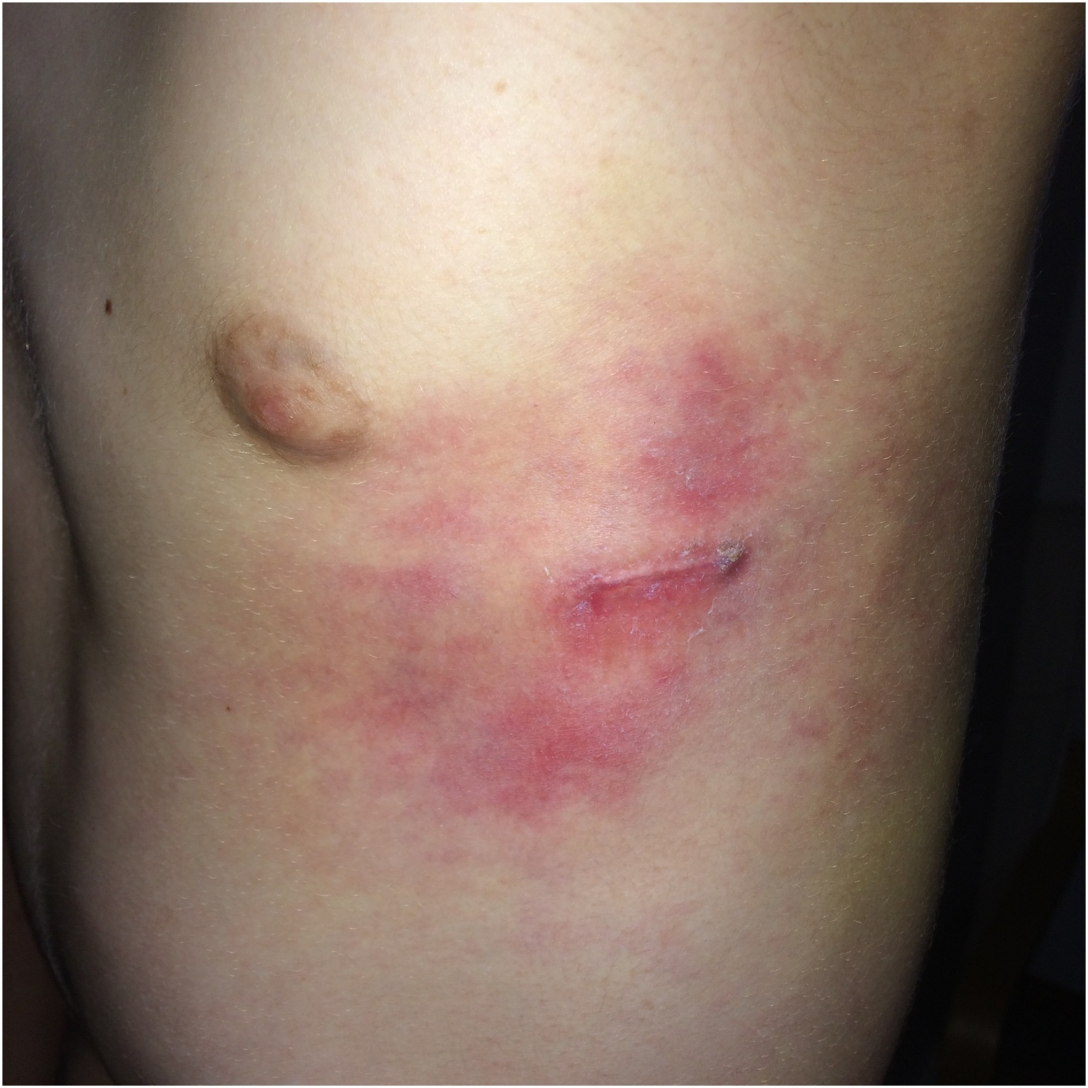
Local skin reaction on left side of the chestwall few months after MIRPE.

One patient developed severe irritative symptoms few weeks after MIRPE with a rash (Fig 6), severe pain, and lassitude. His therapy included 50mg prednisolone and 1.8g clindamycine daily without improvement of his symptoms. Compared to preop, his urine levels for chromium and nickel were elevated four weeks after MIRPE above the reference values, while his blood values were normal. After five months, he had elevated nickel levels in blood and urine as well as increased chromium levels in urine. Due to the severity of his symptoms, we indicated bar explantation five months after MIRPE. His condition improved quickly after removal.

## Discussion

A few studies have already shown that there is trace metal release after MIRPE and that this could be problematic. Cundy et al. showed significantly elevated serum chromium levels 13 months post MIRPE [6]. Torre et al. found, moreover, that chromium levels in blood more frequently increased when patients had multiple bars *(p = 0*.*05)* as well as stabilizers *(p = 0*.*03)* [7]. One group examined trace metal accumulation in hair and found a significant increase of nickel and chromium after MIRPE, with a higher increase in the double-bar group [15]. We have previously also described a significant release of nickel and chromium in local tissue at bar removal and, in addition, increased trace metals in blood and urine [5]. To the best of our knowledge, no published study has, to date, evaluated the kinetics and clearance of systemic trace metal contamination in patients undergoing MIRPE.

The results of the current prospective study show that nickel and chromium ions in blood and urine increased in the early postoperative period and reached significance one year post MIRPE. All reference values were exceeded after one year, implying a considerable trace metal contamination in these patients. In the late follow-up after bar removal, we could demonstrate that metal levels decreased, except nickel in blood, which was still significantly elevated. This finding demonstrates that patients sustain a prolonged metal contamination.

Based on studies of metal-on-metal hip arthroplasty, it is known that metal contamination can cause, amongst other things, local tissue reaction, pseudotumors, and implant failure [8, 11]. In our collective, we saw, amongst other things, a local rash and swelling as acute problems triggered by the metal contamination. In the first five weeks, when these reactions occurred, we could show increased systemic trace metal values, although they did not reach statistical significance. Nonetheless, the tissue trace metal contamination has to be much higher at this early timepoint because it is directly exposed to the stainless steel, leading to these local reactions. In the other reports, the irritative reactions occurred in a similar timeframe of up to 40 days after MIRPE [2, 16]; however, two other groups reported allergic reactions up to nine months after MIRPE [17, 18]. Release of trace metal ions at a slower pace could lead to these late reactions. Some patients of our cohort had an asymptomatic course despite extremely high trace metal values. Therefore, the irritative reactions appear to be due to individual sensitivity to trace metal exposure. This finding is in alignment with the report that metal ion release does not necessarily lead to metal allergy [12].

Clearance of chromium blood levels after bar removal was slower in our cohort than published data from orthopedics. After removal of a metal-on-metal hip replacement, the chromium blood values showed an average decline of 1% per day [19, 20]. In patients with ultra-high chromium levels, however, elevated chromium values were still found one year later [19–21]. These results show that it takes time for chromium values to reach normal levels. Nickel levels in blood were increasing in the further course after bar removal in our patient group. However, urine levels showed a decline of nickel and chromium of 71% and 61% one year after bar removal. It is not clear what caused the different kinetics of nickel in blood and urine after bar removal. One possible explanation is the fact that trace metals, released by steel implants, are mostly excreted rapidly via urine, but in the further course the remaining trace metals cannot be eliminated via renal or intestinal pathways and remain in the blood circulation. Additionally, trace metals can be stored in different tissues and organs and be reabsorbed into the blood over time [22, 23].

The effect of this long-term nickel exposure is unknown. Generally, little is known about potential long-term consequences of trace metal contamination, as its biological effects and potential toxicity are poorly understood [11, 24]. In particular, the biological effects of an internal exposure to chromium after a steady release from an articulating implant have rarely been investigated [25]. In patients undergoing hip arthroplasty, tissue oxidative damage and chromosomal aberrations have been found after chromium contamination [24, 26]. One group demonstrated a correlation of chromium concentration and chromosomal damage in vitro [27], whereas others failed to show a correlation [24]. Nickel contamination may have a carcinogenic effect and can also result in damage to the immune system and DNA [28]. These would be significant consequences, therefore, long-term nickel contamination after MIRPE and its potential long-term consequences need further investigation.

As we have shown, individual sensitivity to metals is an important factor. To prevent implantation of a pectus bar in patients that have already developed a metal allergy, testing is recommended in high-risk patients, namely female patients and patients with a personal or family history of metal allergy [29]. A patch test with metal salts should be used instead of a manufacturer-supplied stainless-steel alloy because the nickel is more tightly bound in the disk, preventing its release and leading to a low bioavailability [17, 30]. To clarify indeterminate patch test results, a lymphocyte transformation test could be applied with a higher sensitivity [13]. Nonetheless, the complex immunologic environment in tissue to which the implanted bar is exposed might cause a hypersensitivity reaction that is not displayed by an epicutaneous test [30]. Therefore, some patients mentioned in published reports developed irritative symptoms after metal exposure despite a negative preoperative patch test [2, 3, 16, 31].

One limitation of our study is the small sample size. Furthermore, single-time-point urine collection could result in an uncertainty due to possible day-to-day and within-day variances. Additionally, we could not measure the individual intake of nickel and chromium via food, water, or other consumer products. Sample collection between surgeries and after bar removal could not be obtained from all patients. The low number of patients analyzed after bar removal could result in a bias, but the fact that symptomatic as well as asymptomatic patients were included in sample collection suggests a representative picture. To lower these possible biases, we performed an intra-individual analysis within this longitudinal study.

## Conclusions

In conclusion, patients undergoing MIRPE with a stainless-steel bar are rapidly exposed to increased local and even systemic nickel and chromium levels. Elevated metal levels in combination with irritative symptoms suggest metal intoxication. After bar removal, mean nickel levels in blood were still increasing, while all other parameters decreased. Long-term consequences cannot be reliably estimated so far and need to be evaluated in further studies. Minimization of trace metal release after MIRPE should be a priority in its advancement.
